# A Selective Cell Population from Dermis Strengthens Bone Regeneration

**DOI:** 10.5966/sctm.2015-0426

**Published:** 2016-08-08

**Authors:** Tingliang Wang, Jinguang He, Yang Zhang, Wenjun Shi, Jiasheng Dong, Ming Pei, Lian Zhu

**Affiliations:** ^1^ Department of Plastic and Reconstructive Surgery, Shanghai Ninth People's Hospital Affiliated with Shanghai Jiao Tong University School of Medicine, Shanghai, People's Republic of China; ^2^ Stem Cell and Tissue Engineering Laboratory, Department of Orthopaedics, and Division of Exercise Physiology, West Virginia University, Morgantown, West Virginia, USA

**Keywords:** Skin, Bone morphogenetic protein receptor type IB, Bone tissue engineering, Coral, Calvarial defect

## Abstract

Finding appropriate seed cells for bone tissue engineering remains a significant challenge. Considering that skin is the largest organ, we hypothesized that human bone morphogenetic protein receptor type IB (BmprIB)+ dermal cells could have enhanced osteogenic capacity in the healing of critical‐sized calvarial defects in an immunodeficient mouse model. In this study, immunohistochemical staining revealed that BmprIB was expressed throughout reticular dermal cells; the positive expression rate of BmprIB was 3.5% ± 0.4% in freshly separated dermal cells, by flow cytometry. Furthermore, in vitro osteogenic capacity of BmprIB+ cells was confirmed by osteogenic‐related staining and marker gene expression compared with unsorted dermal cells. In vivo osteogenic capacity was demonstrated by implantation of human BmprIB+ cell/coral constructs in the treatment of 4‐mm diameter calvarial defects in an immunodeficient mouse model compared with implantation of unsorted cell/coral constructs and coral scaffold alone. These results indicate that the selective cell population BmprIB from human dermis is a promising osteogenic progenitor cell that can be a large‐quantity and high‐quality cell source for bone tissue engineering and regeneration. Stem Cells Translational Medicine *2017;6:306–315*

**Significance Statement**

Dermal cells are a promising cell population for bone regeneration; unfortunately, the osteogenic potential of unsorted cells was quite low. This study demonstrated that a specific cell population in human dermis—bone morphogenetic protein receptor IB (BmprIB)+ cells—exhibited significantly higher osteogenic potential than unsorted dermal cells by repairing critical‐sized calvarial defects in an immunodeficient mouse model. This animal study is an extension of a previous in vitro finding in which BmprIB was proven as a marker for enrichment of osteogenic precursor‐like cells in human dermis, indicating that the selective cell population BmprIB from human dermis is a promising osteogenic progenitor cell that can provide a large‐quantity and high‐quality cell source for bone tissue engineering.

## Introduction

Large bone defects are clinically challenging. Bone tissue engineering is a promising approach for bone regeneration 1 in which bone marrow stromal cells (BMSCs) are a well‐studied and commonly used type of therapeutic mesenchymal stem cell (MSC) 2, 3. Their osteogenic potential is proven but they are difficult to harvest and have a low proliferation rate, which limits their application. Other potential candidates include adipose stem cells (ASCs) and induced pluripotent stem cells 4; however, optimum conditions for application of those cells are not yet established. Despite efforts through the years and the substantial progress that has been made, the ideal seed cells have still not been determined. We theorize that sources that are easily accessible and have an ample number of cells as well as strong osteogenic differentiation capacity may be the key to the current dilemma.

Skin, the largest organ of the human body, consists of the epidermis, dermis, and appendages, such as hair follicles. Adult multipotent stem cells have been identified from all skin locations, such as stem cells from epidermis 5, 6, dermis 7 8 9, and hair follicle stem cells from appendages 10. Despite being a large potential cell source for regeneration, stem cells from skin tissue have a limited capacity for osteogenesis 8, 11, 12. Our recent study demonstrated that the isolation of a specific cell population with expression of bone morphogenetic protein receptor type IB (BmprIB) could dramatically enhance dermal cells’ in vitro osteogenic capacity 11. In this study, we hypothesized that human BmprIB+ dermal cells could have enhanced osteogenic capacity in the healing of critical‐sized calvarial defects in an immunodeficient mouse model.

## Materials and Methods

### Dermal Cell Culture and Sorting

Foreskin samples were obtained from routine circumcision of children aged 6 months to 12 years of age (*n* = 8) following approval of the Ethics Committee of Shanghai Jiaotong University School of Medicine; informed written consent was provided by the parents. The foreskin specimen was depleted of subcutaneous tissue and cut into approximately 5 mm × 2 mm pieces. Next, the specimen was incubated in 8 U/ml Dispase (Worthington Biochemical, Lakewood, NJ, http://www.worthington-biochem.com) at 4°C overnight. The dermis was separated from the epidermis, cut into small pieces, and further digested in 2 mg/ml collagenase (NB4 [PZ activity, 0.170 U/mg]; SERVA Electrophoresis, Germany, http://www.serva.de), which was diluted in Dulbecco's modified Eagle's medium–low glucose (DMEM‐lg) (Thermo Fisher Scientific Life Sciences, Waltham, MA, http://www.thermofisher.com) at 37°C for 3 hours in a shaking water bath. The cell suspensions were filtered through a 40‐μm cell strainer (BD Biosciences, Franklin Lakes, NJ, http://www.bd.com/) and either processed for magnetic‐activated cell sorting (BmprIB+ cells) or directly placed in 10‐cm culture plates (unsorted dermal cells [usDCs]) with complete medium containing DMEM‐lg supplemented with 10% fetal bovine serum (FBS) (Thermo Fisher), 100 U/ml penicillin, and 100 μg/ml streptomycin (all from Sigma‐Aldrich, St. Louis, MO, https://www.sigmaaldrich.com) at a density of 1 × 10^5^/cm^2^.

For magnetic‐activated cell sorting, the cell suspensions were centrifuged and resuspended in phosphate‐buffered saline (PBS; Sigma‐Aldrich) containing 0.5% bovine serum albumin (BSA; Sigma‐Aldrich), labeled with phycoerythrin (PE)‐conjugated anti‐human BmprIB antibody (FAB5051P; R&D Systems, Minneapolis, MN, https://www.rndsystems.com), and further incubated with anti‐PE microbeads (catalog no. 130‐048‐801; Miltenyi Biotec, Bergisch Gladbach, Germany, http://www.miltenyibiotec.com). The BmprIB+ cells were obtained as the incubated cell suspensions passed through the Miltenyi Biotec AutoMACS device, according to the manufacturer's instructions. Briefly, freshly isolated dermal cells were incubated in an incubation buffer (PBS containing 0.5% BSA) containing PE‐conjugated anti‐human BmprIB antibody (R&D Systems) for 60 minutes, followed by incubation with anti‐PE microbeads (Miltenyi Biotec) for 15 minutes. The incubation process was conducted on ice. Cells were passed through a 40‐μm cell strainer before running the AutoMACS device. The obtained cells were plated in complete medium at 37°C, 5% CO_2_, with medium changed after 24 hours to remove nonadherent cells. Cells were grown in medium that was changed every 3 days until they reached 80% confluence. They were then trypsinized and passaged.

For localization of BmprIB+ cells in the dermis, freshly obtained human foreskin samples were fixed in 4% paraformaldehyde (Sigma‐Aldrich), dehydrated in graded ethanol solutions, and paraffin embedded. Immunohistochemical staining was performed by using a primary antibody against human BmprIB (catalog no. ab78417; Abcam, Cambridge, MA, http://www.abcam.com) and processed by following the manufacturer's protocols to localize BmprIB+ cells. Briefly, the sections were incubated with an anti‐human BmprIB antibody (Abcam) at 4°C overnight, followed by incubation with horseradish peroxidase‐conjugated goat anti‐mouse IgG antibody (Sigma‐Aldrich) at 4°C for 30 minutes.

To determine the percentage of BmprIB+ cells in the dermis, flow cytometric analysis of cell suspensions was performed using PE anti‐human BmprIB antibody according to the manufacturer's instructions. Briefly, the cells were incubated with PE anti‐human BmprIB antibody in a cytometry buffer (0.5% BSA, 0.05% azide in PBS) for 60 minutes, then washed, centrifuged, and resuspended. Finally, analysis was performed on a flow cytometer instrument (Beckman Coulter, Miami, FL, https://www.beckmancoulter.com).

### Evaluation of Cell Proliferation and Osteogenic Differentiation

The Alamar Blue assay (Thermo Fisher) was performed in triplicate to measure the proliferation and viability of the BmprIB+ cells (BmprIB) according to the manufacturer's protocol. In brief, the BmprIB+ cells and usDCs were placed into 96‐well plates (BD Biosciences) at 2 × 10^3^ cells per well at passage 2 and incubated in the medium with 10% Alamar Blue reagent for 24, 48, 72, and 96 hours. Culture supernatants were transferred to 96‐well plates and quantified spectrophotometrically for absorbance with a microplate reader (Safire; Tecan Trading, Mannedorf, Switzerland, http://www.tecan.com) at wavelengths of 570 and 600 nm.

Sorted and unsorted cells were induced in osteogenic medium containing complete medium supplemented with dexamethasone (10^−8^ M), β‐phosphoglycerol (10 mM), and ascorbic acid (50 mg/L) (all from Sigma‐Aldrich) after reaching 80% confluence at passage 2. Osteogenic differentiation was evaluated by alkaline phosphatase (ALP) staining at day 7 and alizarin red S (ARS) staining for calcium nodules at day 28, as well as quantitative real‐time polymerase chain reaction (PCR) for osteogenic marker gene expression of *ALP*, osteocalcin (*OCN*), osteopontin (*OPN*), and bone sialoprotein (*BSP*) in triplicate experiments. Briefly, RNA from cell samples was prepared with Trizol (Thermo Fisher ) and cDNA was synthesized from RNA using reverse transcriptase (TaKaRa Biotechnology, Otsu, Japan, http://www.takara-bio.com). Real‐time PCR was performed in an ABI 7300 real‐time PCR system (Thermo Fisher) using the SYBR Premix Ex Tag kit (TaKaRa Biotechnology). The following primers were used: *β‐actin*: forward, 5′‐CATCTCTTGCTCGAAGTCCA‐3′ and reverse, 5′‐ATCA TGTTTGAGACCTTCAA‐3′; human *ALP*: forward, 5′‐TACAAGCACTCCCACTTCATC‐3′ and reverse, 5′‐AGACCCAATAGGTAGTCCACAT‐3′; human *OCN*: forward, 5′‐CTCACACTCCTCGCCCTATT‐3′ and reverse, 5′‐ CCCAGCCATTGATACAGGTAG‐3′; human *OPN*: forward, 5′–CATGAGAATTGCAGTGATTTGCT‐3′ and reverse 5′‐CTTGGAAGGGTCTGTGGGG‐3′; and human *BSP*: forward, 5′‐ TGCAATCCAGCTTCCCAAGA‐3′ and reverse 5′‐ TTGACGCCCGTGTATTCGTAC‐3′.

### Characteristics of Coral Scaffolds and Cells Seeded on Scaffolds

The coral used in this study was obtained from Hainan, China. The coral had porosity of 59.5% ± 7.0%, a mean pore diameter of 180 ± 55 μm, and wall thickness of 88 ± 26 μm. Natural corals were manufactured into wafers 4 mm in diameter by 1‐mm thick; they were washed ultrasonically and autoclaved before they were used as scaffolds in the following experiments. Microcomputed tomography (µCT; μCT80; Scanco Medical, Basserdorf, Switzerland, http://www.scanco.ch) was performed to characterize the coral structure.

For the preparation of premature tissue constructs, both sorted and unsorted cell suspensions (at a density of 4 × 10^7^ cells/mL) at passage 2 were seeded on coral wafers at a density of 2 × 10^5^ cells per scaffold (approximately 1.5 × 10^7^ cells per cm^3^). The suspensions of 5 μl of cells per scaffold were carefully pipetted onto the scaffolds in 6‐well plates and incubated for 1 hour to allow for cell attachment before the remainder of the culture medium was added to cover the cell/scaffold complexes. The scaffolds were incubated in complete medium for 3 days before they were transferred into osteogenic medium in 6‐well plates for 21‐day static culture, with medium change every 3 days.

For cell proliferation assays on the coral scaffolds, sorted and unsorted cells were seeded onto scaffolds in 24‐well plates at a density of 5 × 10^3^ cells per scaffold in triplicate. Coral scaffolds were incubated in complete medium with 10% Alamar Blue reagent for 12, 24, 36, 48, 60, 72, 84, and 96 hours. Culture supernatants were transferred to 96‐well plates and spectrophotometrically quantified for absorbance with a microplate reader (Tecan Trading) at wavelengths of 570 and 600 nm. Nonseeded coral scaffolds incubated in complete medium were used as blank controls.

Scanning electronic microscopy (SEM; Philips XL‐30; Philips, Amsterdam, The Netherlands, http://www.philips.com) was used to determine the adhesion and growth of BmprIB+ cells on the scaffolds at days 1, 3, and 7. After cell seeding at a density of 2 × 10^5^ cells per scaffold in regular culture medium, the constructs were fixed at days 1, 3, and 7 in 2.5% glutaraldehyde, postfixed with 0.1% osmium tetroxide, dehydrated through an ethanol series, dried in a CO_2_ dryer, coated with gold, and examined with SEM. The coral scaffold alone was also observed using SEM.

The ALP activity and osteocalcin content were evaluated in the constructs at days 1, 3, 7, 14, 21, and 28 after osteogenic induction. For the ALP activity assay, the cell/coral constructs were washed with PBS, homogenized with Tris buffer (pH 7.4; Sigma‐Aldrich), and ultrasonically disintegrated. The cell lysates were incubated with *p*‐nitrophenol (PNP) phosphate substrate solution and alkaline buffer solution in a 37°C water bath for 15 minutes. The absorbance at 405 nm was measured with a spectrophotometer after 0.05 M NaOH was added to stop the reaction; a standard PNP curve with known concentration was used to determine the unknown samples. The osteocalcin content was also evaluated using a human osteocalcin RIA Kit (Biomedical Technologies, Stoughton, MA) according to the manufacturer's instructions. Briefly, the samples were incubated with 100 μl of antiosteocalcin serum and 100 μl of ^125^I‐labeled osteocalcin antigen for competitive binding reaction at 4°C for 24 hours. After the reaction, 500 μl of separating agent was added to each tube and incubated for 20 minutes at room temperature. The radioactivity (in counts per minute) of the precipitate was measured by a radiation monitoring instrument (B291000; PerkinElmer, Waltham, MA, http://www.perkinelmer.com). The binding rate of each sample was calculated and, based on the standard curve, the osteocalcin content of each sample was subsequently determined.

### In Vivo Evaluation of Efficacy of Calvarial Defect Resurfacing

The animal experimental protocol was approved by the Animal Care and Experiment Committee of Shanghai Jiaotong University School of Medicine. After anesthesia, an incision of approximately 2 cm was made sagittally to expose the parietal calvarium; the periosteum was carefully removed with swabs on the right side of the parietal bones. A hollow drill was applied to create 4‐mm diameter defects, which were demonstrated in previous studies as unlikely to spontaneously heal 13, 14. A total of 36 immunodeficient mice, aged 8 weeks, were randomly divided into three groups. The defects were repaired by BmprIB+ cell/coral constructs in the experimental group, while the other two groups were separately repaired with coral scaffolds alone or usDCs/coral constructs as a control. The cell/scaffold constructs used in animal experiments were incubated in osteogenic medium for 3 weeks before implantation. The incisions were sutured with 5‐0 nylon sutures. µCT (MicroCT80; Scanco Medical) was performed to confirm the surgical effectiveness 24 hours after the operation.

For in vivo osteogenic analysis, histological examinations were performed 6 weeks after surgery. Six mice in each group were euthanized. Harvested parietal bone samples were washed in PBS, fixed in 4% paraformaldehyde for 1 day, decalcified for 2 weeks in 10% ethylenediamine tetraacetic acid disodium salt (Sigma‐Aldrich) solutions, dehydrated in graded ethanol solutions, paraffin embedded, sectioned to 10 μm, and mounted on glass slides. Hematoxylin and eosin (H&E) staining, Masson trichrome staining, and immunohistochemical staining were carried out to evaluate bone formation, structure remodeling, and angiogenesis. The primary antibodies used in immunohistochemical staining were as follows: antiosteopontin (catalog no. ab8448; Abcam), antiosteocalcin (catalog no. ab93876; Abcam), anti‐bone sialoprotein (catalog no. AB1854; Merck Millipore, Guyancourt, France, http://www.emdmillipore.com), and anti‐CD31 (catalog no. ab28364; Abcam).

For hard‐tissue histology, the remaining 6 mice in each group were euthanized 24 weeks postoperatively. Parietal bones were fixed in 4% paraformaldehyde for 1 day, dehydrated in an ethanol series and toluene, and embedded in methyl methacrylate. The blocks were sectioned into 10‐μm slices using a hard‐tissue microtome (Leica, Germany, http://www2.leicabiosystems.com). Goldner trichrome staining was carried out to evaluate bone formation and remodeling: mineralized bone stained green, osteoid stained red, and cartilage stained purple.

Long‐term bone formation was evaluated 24 weeks postoperatively, taking into consideration coral degradation and bone remodeling. Three‐dimensional (3D) reconstruction of the µCT images was performed and the repair percentage of the defects, which was defined as the opaque area over total defect area, was processed using associated software GE AW4.1 (GE Healthcare, Little Chalfont, U.K., http://www3.gehealthcare.com). Detailed evaluation of bone structural parameters determined by µCT analysis was also carried out, including bone volume (BV), bone volume fraction (bone volume per total volume [BV/TV]), trabecular number (Tb.N), trabecular thickness (Tb.Th), trabecular spacing (Tb.Sp), and connect density (Conn.D).

### Statistical Analysis

Numerical data are presented as the mean and the standard deviation of the mean. All statistical analyses were performed with SPSS 13.0 (IBM, Chicago, IL, http://www.ibm.com). Statistically significant differences of three groups were determined using one‐way analysis of variance and Tukey test. Other data between the two groups in the current study were assessed using Student's *t* test. A *p* value less than .05 was considered statistically significant.

## Results

### Characterization of BmprIB+ Dermal Cells

**Figure 1:**
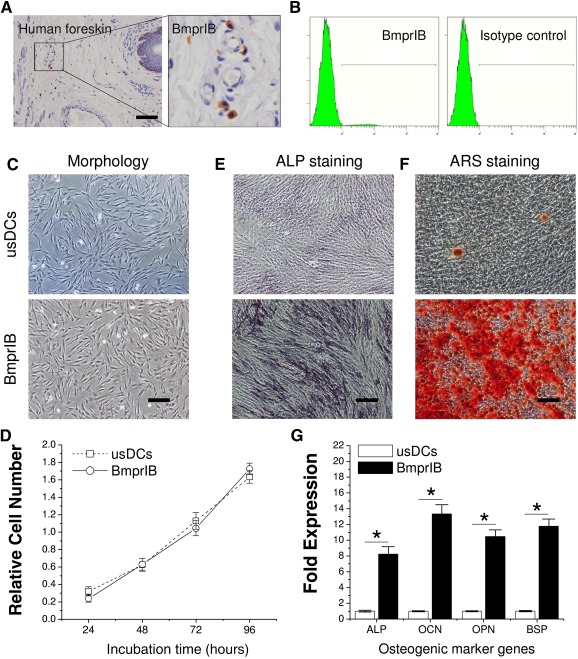
Characterization of BmprIB+ dermal cells. **(A):** Immunohistochemical staining of BmprIB in human foreskin. **(B):** Percentage evaluation of BmprIB expression in freshly isolated human dermal cells by flow cytometry. Histograms of BmprIB expression (left; 3.5% ± 0.4%) and isotype control (right). **(C):** Cell morphology under phase‐contrast microscopy. **(D):** The proliferative potential was assessed with the Alamar Blue assay in usDCs and BmprIB+ cells. Cell numbers were quantified by the absorbance wavelength at 570 nm using a spectrophotometer at 24, 48, 72, and 96 hours. Data represented the mean of three individuals and each of them was the mean of triplicate experiments. The osteogenic potential of BmprIB+ cells was demonstrated by ALP staining **(E)** and ARS staining **(F)**. **(G):** The mRNA expression levels of *ALP* (at day 7) and *OCN*, *OPN*, and *BSP* (at day 21) were analyzed by real‐time polymerase chain reaction after osteogenic induction. Data represent the mean ± SD (*n* = 3). ∗, *p* < .05 indicates statistical significance. Scale bars = 50 µm. Abbreviations: ALP, alkaline phosphatase; ARS, alizarin red S stain; BmprIB, bone morphogenetic protein receptor type IB; usDC, unsorted dermal cell.

To determine the site and percentage of BmprIB+ dermal cells, the data from in situ analysis of human foreskin paraffin sections by immunohistochemical staining revealed that BmprIB was present on separate dermal single cells, mostly in the reticular layer, rarely in the papillary layer, and with a perivascular expression pattern (Fig. 1A). A total of 6 foreskin single‐cell suspension samples (each sample in triplicate) were tested using flow cytometry, showing an expression rate of 3.5% ± 0.4% (range, 2.8%–3.9%) (Fig. 1B).

Phase‐contrast microscopy showed that the BmprIB+ cells obtained by magnetic‐activated cell sorting exhibited a homogeneous spindle‐shaped morphology, whereas the usDCs had a relatively heterogeneous morphology (Fig. 1C). A cell proliferation assay demonstrated that both sorted and unsorted cells had comparable proliferative capacity during the test period of 96 hours (Fig. 1D).

To determine whether cell sorting using BmprIB promoted dermal cell osteogenic potential, ALP staining at day 7 and ARS staining at day 28 were performed to confirm the osteogenic potential after osteogenic induction. ALP activity (Fig. 1E) for early detection of osteogenesis and calcium deposition for late detection of osteogenesis (Fig. 1F) in BmprIB+ cells were more intense than in the usDCs group. The expression level of osteogenic‐specific genes, including *ALP*, *OCN*, *OPN*, and *BSP*, confirmed that BmprIB+ cells exhibited a dramatically enhanced osteogenic capacity compared with unsorted cells (Fig. 1G).

### Dermal Cell/Coral Scaffold Compatibility

**Figure 2:**
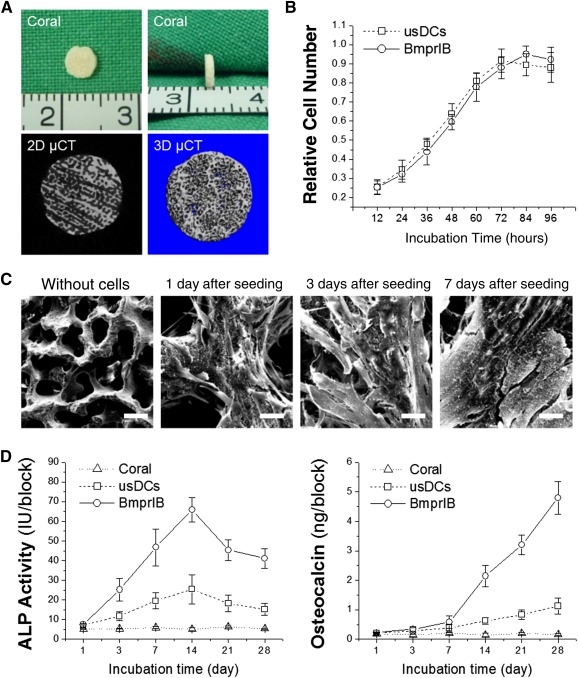
Dermal cell/coral scaffold compatibility. **(A):** Gross view of a coral scaffold with the size of 4‐mm diameter by 1‐mm thickness (top panel); two‐dimensional (left) and three‐dimensional images (right) of a coral scaffold by µCT scanning (bottom panel). **(B):** The cell proliferation analysis of the BmprIB+ cells and usDCs on the coral scaffold was assessed with the Alamar Blue assay at 12, 24, 36, 48, 60, 72, 84, and 96 hours. Data represent the mean of three individuals and each of them was the mean of triplicate experiments. **(C):** Scanning electron microscopy evaluation of the adherence and matrix deposition of BmprIB+ cells on the coral scaffold 1, 3, and 7 days after seeding. Scale bar = 20 µm. The coral alone without cell seeding served as a control. Scale bar = 100 µm. **(D):** Osteogenic potential of BmprIB+ cells and usDCs on the coral scaffold was evaluated according to the ALP activity and osteocalcin content at days 1, 3, 7, 14, 21, and 28 after osteogenic induction, respectively. Data represent the mean of three individuals and each of them was the mean of triplicate experiments. Abbreviations: 2D, two‐dimensional; ALP, alkaline phosphatase; BmprIB, bone morphogenetic protein receptor type IB; μCT, microcomputed tomography; usDC, unsorted dermal cell.

As shown in Figure 2A, the customized coral scaffold was 4 mm in diameter by 1‐mm thick. Proliferative data indicated that both sorted and unsorted cells on coral scaffolds exhibited similar exponential growth curves (Fig. 2B). SEM observation showed that dermal cells attached to the coral scaffold maintained a spindle‐shaped morphology after seeding for 1 day and continued to secrete abundant extracellular matrix (ECM) at days 3 and 7 (Fig. 2C). During 28‐day osteogenic induction, ALP activity in sorted and unsorted cells on coral scaffolds kept increasing after seeding until reaching a peak at around day 14 and then began to decrease thereafter, particularly for the BmprIB group (*p* < .05) (Fig. 2D). In contrast, the late‐stage osteogenic marker osteocalcin continued to increase during the entire detection period of 28 days in both groups; there was a rapid increase after the test point of day 7 in the BmprIB group. However, the usDCs group remained at a low level and increased slightly over time (*p* < .05) (Fig. 2D).

### Evaluation of Early‐Stage Osteogenic Reconstruction

**Figure 3:**
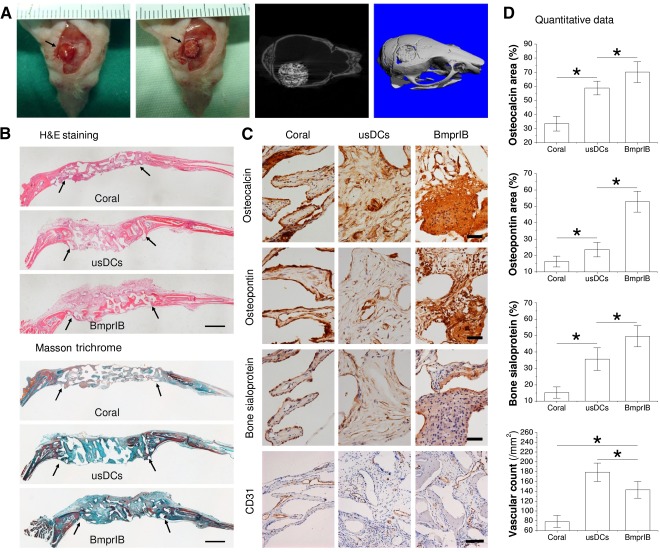
Evaluation of early‐stage osteogenic reconstruction. **(A):** A full‐thickness defect 4 mm in diameter was created by a hollow drill on the right side of the calvarial bone and repaired by coral alone, usDCs/coral, or BmprIB+ cells per coral in each group (top panel). Representative images of two‐dimensional (left) and three dimensional (right) µCT scanning were taken 24 hours after the surgery (bottom panel). **(B):** Histological analyses including H&E staining and Masson trichrome staining were used to evaluate new bone formation 6 weeks postimplantation. The black arrows indicate both ends of the defect. Scale bar = 1 mm. **(C):** Immunostaining was used to evaluate osteogenic marker expression, including osteocalcin, osteopontin, bone sialoprotein, and vascular marker CD31 in newly formed bone. Scale bar = 50 µm. **(D):** Histomorphometric analysis was used to quantify the percentage of positive staining area or vascular count. Data represent the average ± SD (*n* = 6). ∗, *p* < .05 indicates statistical significance. Abbreviations: BmprIB, bone morphogenetic protein receptor type IB; H&E, hematoxylin and eosin; usDC, unsorted dermal cell.

The surgical procedures and animal model (Fig. 3A) appeared to be effective; none of the experimental mice experienced death, infection, or delayed healing of incisions in the early postoperative stage. With a long observation period of 24 weeks, the mice all survived without complications, such as seizures, weight loss, or deformity.

To investigate premature tissue constructs implanted in vivo, histological analyses were used to evaluate viability, osteogenesis, and angiogenesis 6 weeks postsurgery. Both H&E staining and Masson trichrome staining (Fig. 3B) revealed that the coral scaffold with cell seeding exhibited much denser tissue for repairing the defect than coral alone. Coral scaffolds with usDCs or BmprIB+ cells degraded much faster than coral alone, suggesting that cells seeded into the scaffold could accelerate scaffold resorption. Moreover, initial bone formation was observed in the BmprIB group, whereas osteoid was minimally observed in the other two groups. Immunostaining was used to detect critical ECM proteins for osteogenesis, including osteocalcin, osteopontin, and bone sialoprotein. The expression of these matrix proteins was intensively stained in the BmprIB group compared with the other two groups (Fig. 3C), which was confirmed by quantitative data (Fig. 3D). To further evaluate the angiogenesis of the scaffold, the surface marker CD31, which was used as a marker of blood vessel endothelial cells, was examined. Quantitative histomorphometric analysis of CD31 demonstrated that angiogenesis in the usDCs group was higher than in the BmprIB group and coral‐alone group (Fig. 3D). The results also inferred that the coral scaffold was accessible for blood vessels to grow into and could supply sufficient nutrition for bone formation.

### Evaluation of Late‐Stage Osteogenic Reconstruction

**Figure 4:**
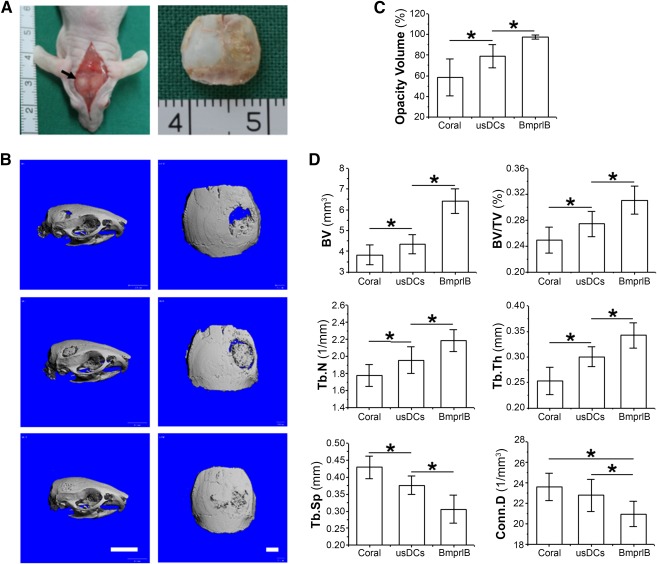
Microcomputed tomography (µCT) evaluation of late‐stage osteogenic reconstruction. **(A):** The defect was repaired by engineered bone on the right side of the mouse cranium 24 weeks postimplantation. Black arrows mark the repaired defect. **(B):** Representative three‐dimensional µCT images of parietal bones from each group 24 weeks postimplantation. Scale bar = 5 mm (left) and 1 mm (right). **(C):** The opaque percentage of each group was evaluated. **(D):** Other important bone structure parameters, such as BV (mm^3^), BV/TV ratio (%), the Tb.N (1/mm), the Tb.Th (mm), the Tb.Sp, and Conn.D, were compared among these three implant groups with coral, usDCs/coral, and BmprIB/coral. Data represent the mean ± SD (*n* = 6). ∗, *p* < .05 indicates statistical significance. Abbreviations: BmprIB, bone morphogenetic protein receptor type IB; BV, bone volume; BV/TV, ratio of bone volume to total volume; Conn.D, connectivity density; Tb.N, number of trabeculae; Tb.Sp, trabecular spacing; Tb.Th, thickness of the trabecular structure; usDC, unsorted dermal cell.

The mice were euthanized 24 weeks after surgery. The tissue samples (Fig. 4A) were evaluated by µCT scanning and histology analysis. 3D reconstruction of µCT images demonstrated that a nearly 100% opacity volume repair was achieved in the BmprIB group. Coronal plane reconstruction showed that a bony connection was formed between the circular defect and the normal bone edge, and the thickness of the engineered bone fit the cranium. In contrast, the two control groups (usDCs and coral alone) were relatively inferior (Fig. 4B).

The findings were supported by quantitative data. The repair percentage of the BmprIB group was as high as 97.4% ± 2.1%, whereas that in the usDCs group was 78.7% ± 11.3% and the coral‐alone group was 58.3% ± 17.6% (*n* = 6; *p* < .05) (Fig. 4C). Other important bone structure parameters (Fig. 4D), such as BV (mm^3^),BV/TV ratio (%), the Tb.N (1/mm), and the Tb.Th (mm), showed a similar trend as opacity volume (%) (Fig. 4C). The Tb.Sp and Conn.D of the BmprIB group were smaller than that of the other two groups (Fig. 4D), indicating that the matrix deposition and structure remodeling were persistent in the formation and maturation of engineered bone.

**Figure 5:**
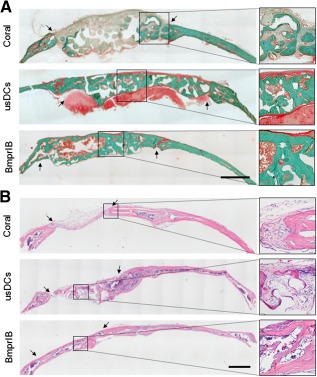
Histological evaluation of late‐stage osteogenic reconstruction. **(A):** Goldner trichrome staining was used for plastic‐embedded, undecalcified, 24‐week regenerated bone. Mature bone matrix stained green and immature new bone matrix stained red. **(B):** Hematoxylin and eosin staining was also used to evaluate 24‐week regenerated bone. The black arrows indicate both ends of the defect. Scale bar = 1 mm. Abbreviations: BmprIB, bone morphogenetic protein receptor type IB; usDC, unsorted dermal cell.

Hard‐tissue sections stained by Goldner trichrome were used to evaluate bone formation and remodeling at 24 weeks. The defects were completely repaired by mature bone in the BmprIB group and partially repaired by osteoid in the usDCs group, whereas osteoid tissue was unremarkable in the coral‐alone group (Fig. 5A). Besides the mature bone, which stained green, the typical structure of regenerated marrow cavity was observed in the BmprIB group. The joint between the repaired and normal bone was connected by compact bony union in the BmprIB group. H&E staining demonstrated that the defects in the BmprIB group were repaired by a structure resembling diploic bone with dense inner and outer layers (Fig. 5B). On the contrary, only fibrous tissue that stained light red was found in the coral‐alone group, whereas bone and fibrous tissue filled in the defects in the usDCs group (Fig. 5B).

## Discussion

Dermal cells are a promising cell population for bone regeneration; unfortunately, the osteogenic potential of unsorted cells is quite low 8, 11, 12. To our knowledge, this is the first study that demonstrated that a specific cell population in human dermis, BmprIB+ cells, exhibited significantly higher osteogenic differentiation potential than unsorted dermal cells by repairing critical‐sized calvarial defects in an immunodeficient mouse model. This animal study is an extension of our previous in vitro finding in which BmprIB was proven to be a marker for enrichment of osteogenic precursor‐like cells in human dermis 11. This was also supported by a recent report in which, compared with the unsorted cells, BmprIB+ human ASCs were found to have an enhanced ability to form bone both in vitro and in vivo 15, suggesting that positive selection for BmprIB+ and manipulation of the bone morphogenetic protein (Bmp) pathway in these cells may yield a highly osteogenic subpopulation of cells for bone tissue engineering.

It is well‐known that the Bmp/Bmpr pathway plays an important role in osteogenesis 16 17 18. BmprIB as a subtype of the Bmp receptors (subtypes IA, IB, and II) is reported to play an important role in Bmp signal transduction 16, 17, 19. The BmprII phosphorylates the BmprI after the BmprI and BmprII form hetero‐oligomeric complexes when Bmps bind to the receptors. Subsequently, through the Smad1/5/8 and Smad4 signaling pathways, the final signal is translocated to the nucleus for the regulation of gene transcription 19 20 21. Although the precise role of a selective Bmpr in cell differentiation has not been well established, the subtype BmprIB tends to mediate signals responsible for osteoblast differentiation, whereas BmprIA prefers adipogenic differentiation 17, 19, 22. Zhang et al. found that misexpression of BmprIB caused digit malformation in mouse limb 23. Moreover, the BmprIB signal pathway is essential to the early osteoblast differentiation of human bone cells; downregulating BmprIB expression would significantly reduce ALP activity 16, 20. These findings indicate that cell sorting using BmprIB would benefit cell‐based bone regeneration.

The anatomical niche of BmprIB+ cells in the dermis has not been completely defined. The heterogeneous fibroblast lineages of the dermis, as described by Driskell et al. 24, demonstrated that fibroblasts in the upper and lower dermis were of different origins and had significantly diverse functions. One formed the upper dermis, including the dermal papillae that regulate hair growth and the arrector pili muscle, which controls piloerection. The other forms the lower dermis, including the reticular fibroblasts that synthesize the bulk of the fibrillar ECM, and the preadipocytes and adipocytes of the hypodermis. In situ analysis of skin cryosections by immunohistochemical staining revealed that MSC markers (CD73, CD90, CD105, CD271, and stage‐specific embryonic antigen‐4) are expressed on different dermal cell types. CD73, CD90, and CD105 show similar single‐cell, vascular, and perivascular expression patterns 25. Our data found that BmprIB+ cells were a group of cells with homogeneous origin that resided in the lower dermis and rarely in the upper dermis. The localization also implied that BmprIB+ cells may be related to pericytes, which had been described in many organs as the niche for MSCs 26, 27.

Coral is frequently used as a substitute for treating bone defects. As a natural material, the porous structure and inorganic calcium compounds make it a potential candidate for bone tissue engineering because these properties benefit cell adhesion and proliferation as well as complete degradation in vivo 28 29 30. The degradation rate of coral is variable. Petite et al. reported that in vivo degradation of coral was completed within 4 months 31, whereas Cui et al. found that the in vivo resorption of coral was as long as 24 weeks and delayed degradation beyond 24 weeks occurred at a high frequency 32. The cutting direction is one of the major reasons that pore direction affects the mechanical properties and causes discrepancy in the absorption rate 33. Other factors include different implantation sites, the health status of experimental animals, and coral species.

In this study, we observed that the coral scaffolds, which were all cut from the same coral block, were mostly absorbed at 24 weeks in vivo; however, a small fraction of intact coral could still be observed, especially in the control group with coral alone. This difference may be due to the minor differences in the cutting direction and, more importantly, the local osteoclast recruitment and activity. Whether BmprIB+ cells and usDCs played a role in osteoclast recruitment and activation was unclear; however, we did notice that the degradation velocities of coral scaffolds in the coral‐alone group were much slower than in the cell‐seeded groups in vivo. As the survival of seed cells in engineered bone greatly relies on vasculature to deliver nutrients, new bone formation is closely associated with adequate neovascularization. In the current study, our data demonstrated that coral scaffolds were suitable for vessel ingrowth when implanted for treatment of calvarial defects.

## Conclusion

Our study demonstrated that BmprIB+ cells isolated from human dermis possess enhanced osteogenic potential in both in vitro and in vivo studies, suggesting that BmprIB+ dermal cells are a promising cell source for bone tissue engineering. Despite the success of BmprIB+ dermal cells in the repair of calvarial defects, several mysteries need to be uncovered in further investigations. First, as the osteogenic potential is not necessary for skin injury repair, the physiological role of BmprIB+ cells in the dermis remains to be defined. Second, precise comparison of BmprIB+ cells with other MSCs, such as BMSCs or ASCs, is essential. Third, an immunodeficient animal model was used in this study to avoid immune issues associated with donor cells, while immunology research on the use of allogeneic BmprIB+ cells as seed cells is of great importance. Fourth, juvenile foreskins were used as a cell source in this study. Considering that the primary candidate patients for most tissue engineering applications are older, a future study will investigate the population of BmprIB+ cells in adult skin biopsies and its potential application for bone defect repair.

The low expression or yield of BmprIB+ cells also exists in human ASCs. McArdle et al. found that freshly isolated ASCs stained for BMPrIB‐PE‐Cy7 demonstrated that 7.9% of the population was BMPrIB+ cells 15. They also proposed that lower‐efficiency cell isolation might be because of the use of a low affinity of the antibody for the BmprIB receptor and an indirect method of staining to isolate these cells 15. Despite no comparable data being available regarding BmprIB+ selection in BMSCs, less than 1:10,000 nucleated cells in bone marrow requires extensive in vitro expansion for clinical application 34. However, conventional monolayer culture often results in replicative senescence in terms of the loss of stem cell proliferation and differentiation capacity 35. Recent studies indicate that decellularized stem cell matrix might solve this concern by providing a 3D microenvironment in which stem cells are greatly expanded; more importantly, the differentiation capacity of expanded stem cells can be maintained or even magnified 36 37 38. This novel strategy to overcome the low yield of BmprIB+ cells will also be investigated in a future study.

## Author Contributions

T.W.: conception and design, collection and assembly of data, data analysis and interpretation, manuscript writing; J.H. and Y.Z.: collection and assembly of data, data analysis and interpretation; W.S.: collection and assembly of data; J.D.: provision of study material; M.P.: manuscript writing, final approval of manuscript; L.Z.: conception and design, manuscript writing, final approval of manuscript.

## Disclosure of Potential Conflicts of Interest

The authors indicated no potential conflicts of interest.
